# The association between involuntary subordination and common mental disorders among men who have sex with men (MSM) in Shanghai, China

**DOI:** 10.1186/s12888-019-2329-7

**Published:** 2019-11-27

**Authors:** Shuxian Zhang, Suping Wang, Zezhou Wang, Ying Wang, Xueqin Jiang, Gang Xu, Yong Cai

**Affiliations:** 1 School of Public Health, Shanghai Jiao Tong University, School of Medicine, No.227, South Chongqing Road, Shanghai, 200025 People’s Republic of China; 2 Shanghai Jiao Tong University Institute of Social Cognitive and Behavioral Sciences, No. 800, Dongchuan Road, Shanghai, 200240 People’s Republic of China

**Keywords:** China, Men who have sex with men, Depression, Anxiety, Involuntary subordination

## Abstract

**Background:**

Involuntary subordination is a mechanism that switches off fighting behaviors when a losing organism is unable to continue in a struggle. The study aim was to investigate the association between involuntary subordination and the common mental disorders of anxiety and depression among men who have sex with men (MSM) in Shanghai, China.

**Methods:**

A cross-sectional study was conducted of 547 MSM in four Shanghai districts. Sociodemographic and psychosocial participant data were collected. Logistic regression was used to assess the association between anxiety, depression, and involuntary subordination.

**Results:**

12.2 and 30.9% Of the MSM demonstrated high levels of anxiety and depression respectively. Univariate analysis showed that involuntary subordination and the involuntary subordination constructs of defeat, social comparison, submissive behavior, and entrapment were associated with anxiety and depression. Multivariate analysis indicated that defeat (*ORm* = 1.091, 95% *CI* = 1.004–1.185) and entrapment (*ORm* = 1.174, 95% *CI* = 1.079–1.278) were significantly associated with anxiety. Defeat (*ORm* = 1.265, 95% CI = 1.166–1.372), social comparison (*ORm* = 1.119, 95% *CI* = 1.061–1.181), entrapment (*ORm* = 1.132, 95% *CI* = 1.047–1.224), and submissive behavior (*ORm* = 0.897, 95% *CI* = 0.825–0.975) were significantly associated with depression.

**Conclusions:**

The findings confirmed an association between anxiety, depression, and involuntary subordination among MSM. These findings could form the basis of a new, integrated, and holistic approach to the identification of high-risk groups and the development of interventions for anxiety and depression among MSM.

## Background

There are much higher rates of depression, anxiety, panic disorders, and substance use disorders in men who have sex with men (MSM) than in heterosexual men [5–7, 26, 35, 36]. Research shows that 60% of MSM in China exhibit symptoms consistent with negative mood, such as extreme tension, depression, anxiety, panic, and loneliness, and 20% of these individuals report previous suicidal attempts. MSM are a sexual minority group and therefore may not easily be accepted into mainstream society. MSM are subject to institutionalized prejudice, social stress, social exclusion, physical violence, physical harassment, rape, incest, and destruction of personal property, which can negatively affect their psychosocial condition [20, 32]. According to minority stress theory, MSM experience disproportionately burdensome degrees of stigma, prejudice, and discrimination. As a result, they are at risk for increased levels of depression, substance use [27, 28]. Studies have shown that mental disorders like depression and anxiety contribute to human immunodeficiency virus (HIV) vulnerability and suicidal ideation [18, 23, 24].

According to social rank theory, involuntary subordination is a mechanism that switches off fighting behaviors when a losing organism is unable to continue in a struggle (thus saving the organism from injury). A subordinate individual may feel helpless, hopeless, inferior, and inadequate and exhibit inhibition of thought. Involuntary subordination leads to a redirection of behavior toward the pursuit of more realistic goals and a new position in the social hierarchy ([38, 17]). However, if individuals are incapable of escaping from a defeating situation or cannot accept defeat, involuntary subordination becomes prolonged and maladaptive, and then manifests as major depression and anxiety disorders ([38, 39, 42]). Social rank theory views depression as a natural consequence of prolonged involuntary subordination. Sturman and Mongrain found that involuntary subordination predicts the recurrence of major depression [45]. Involuntary subordination has also been linked to anxiety disorders. Anxious people may have a defense system dominated by frequent social comparisons and a readiness to submit [11, 12, 48, 49].

Early conceptualizations of involuntary subordination defined it as a latent variable derived from entrapment (feeling stuck or trapped and wanting to escape) and poor social comparisons (i.e., feelings of perceived inferiority) [45]. This definition was later considered too narrow and subsequent research has expanded the concept of involuntary subordination to include other constructs. For example, submissive behavior may be associated with involuntary subordination. Taylor has suggested, on theoretical grounds, that defeat should be considered another indicator of involuntary subordination [47]. Therefore, involuntary subordination can be regarded as a range of interconnected feelings and perceptions, including the perception that one is of lower status than others (unfavorable social comparison), the feeling that one has been defeated by a dominant other (social defeat), the feeling that one has no chance to escape an uncontrollable set of circumstances (entrapment), and the perception that one wishes to avoid conflict (submissive behavior) [1, 2, 11, 13]. A confirmatory factor analysis supported this four-variable model of involuntary subordination [44]. This model provides the basis for a new empirical involuntary subordination measure, which draws on items from questionnaire measures of the four variables (unfavorable social comparison, social defeat, entrapment, and submissive behavior) to evaluate involuntary subordination in a more comprehensive way.

Defeat, entrapment, submissiveness, and perceived low status (unfavorable social comparison) are associated with different types of psychopathology through maladaptive functioning of involuntary subordination [13, 15]. However, there is little research on these associations. It also remains unclear whether involuntary subordination, as it reflects social adaptive functions, is associated with psychological problems in sexual minority groups. Therefore, a new focus is needed on the psychological correlates of MSM. The purpose of this study was to investigate symptoms of depression and anxiety among MSM in metropolitan Shanghai, China, and to explore the role of involuntary subordination in depression and anxiety. The primary study hypotheses were (1) involuntary subordination is related to depression and anxiety status and (2) involuntary subordination constructs (defeat, entrapment, submissiveness, and low perceived status) are associated with depression/anxiety. Guided by previous research findings, we used a combined theoretical and empirical approach to examine these hypotheses and generate recommendations for diagnosis and intervention strategies for anxiety and depression among MSM.

## Methods

### Study population and eligibility criteria

The cross-sectional study was conducted from March to May in 2014 in four districts of Shanghai. With the help of the local Center for Disease Control and Prevention (CDC) and non-governmental organizations (NGOs), we could get easy access to the MSM participants. All participants must be at least 18 years old, biologically male and a and responded “Yes” when asked “Have you ever had anal sex with men in the previous 6 months”. A total of 567 MSM agreed to participate, and 547 participants (response rate of 96%) completed the whole questionnaire.

### Recruitment and procedure

As MSM were usually“hidden” populations, the unique “snowball” sampling technique was adopted to obtain participants [8]. The local CDC and NGOs helped us target 5 to 10 so-called “seeds” that met our inclusion criteria. The “seeds” were asked to recruit MSM from the same sociocultural background with them until an adequate sample was obtained. It has been reported that the prevalence of anxiety or depression is about 20 to 60% among MSM. With an expected rate of 40%, the sample size of 512 MSM is allowed for the calculation of a 95% confidence interval with a precision of +/− 6% and a design effect of 2.

The anonymous face-to-face interviews are divided into two part. First, our outreach interviewers were requested to introduce the goal and procedure of the study as well as the potential risks before the participants signed the informed consent. Then, every participant was asked to complete a self-administered questionnaire in 30 min independently in a private room. Commute expense compensation was given to the participant to increase compliance.

### Sociodemographic variables

Sociodemographic data included age, income, marital status (refers to only heterosexual marriage), employment status, place of residence, education level, self-reported sexual orientation, whether one had voluntary HIV counseling and testing in the past 6 months, and the HIV test results.

### Psychological variables

#### Depression

The Center for Epidemiologic Studies Depression scale (CES-D) was used to measure clinically significant depression symptoms [4, 33]. It is a 20-item Likert-type scale. Participants were asked how often they had experienced depressive symptoms within the past week, rated from 0 (less than a day or never) to 3 (5 to 7 days); higher total scores indicate severer depression (Cronbach’s alpha coefficient = 0.891; range 0–57). A cut-off point of score 22 was adopted based on recent validation research [41].

#### Anxiety

The Generalized Anxiety Disorder 7-item scale (GAD-7) was used to measure generalized anxiety disorder [19, 40]. It is a 7-item likert-type scale. Participants were asked how often they had experienced anxiety symptoms in the past 2 weeks, rated from 0 (not at all) to 3 (nearly every day); higher total scores indicate severer generalized anxiety disorder (Cronbach’s alpha coefficient = 0.922; range 0–21). A cut-off point of score 10 was adopted based on previous research [40].

#### Involuntary subordination

Involuntary subordination was measured using the 32-item Involuntary Subordination Questionnaire (ISQ) [44]. The ISQ comprises four subscales representing the dimensions of defeat, entrapment, perceived social comparison, and submissiveness. Sturman selected the eight items with the highest item–total correlations from the Defeat Scale (DS) [13], the Entrapment Scale (ES) [13], the Social Comparison Rating Scale (SCRS) [1], and the Submissive Behavior Scale (SBS) [14] to develop the ISQ. The following are example items from each scale: DS, “I feel that I have not made it in life” and “I feel that I have lost my standing in the world”; ES, “I am in a situation I feel trapped in”; SCRS, “I feel that I am more confident than other people”; and SBS, “I let others criticize me or put me down without defending myself.” The four scales are rated on response scales that vary from 5 points to 10 points. To create a cohesive scale, the response options for all the items selected for the ISQ were changed to a Likert format, as follows: 1 = strongly disagree, 2 = disagree, 3 = neutral (neither agree nor disagree), 4 = agree, and 5 = strongly agree; higher total scores indicate higher levels of involuntary subordination (Cronbach’s alpha coefficient = 0.915; range 35–136). Higher scores on each subscale indicate higher levels of perceived defeat (Cronbach’s alpha = 0.879; range 8–40), entrapment (Cronbach’s alpha = 0.869; range 8–40), and submissiveness (Cronbach’s alpha = 0.632; range 8–40), and lower levels of perceived social comparison (Cronbach’s alpha = 0.780; range 1–29).

### Statistical analysis

Statistical analysis was performed using SPSS Statistics (version 23.0 for Windows, IBM, Armonk, NY, USA). Baseline descriptive statistics were calculated to summarize sociodemographic characteristics and psychosocial variables. Then, univariate analysis was performed using binary logistic regression to detect the association between sociodemographic variables and depression/anxiety, as well as between each involuntary subordination variables and depression/anxiety. Then, the univariate logistic regression analysis was used to examine the association between depression, anxiety, and involuntary subordination among MSM after adjusting for significant sociodemographic variables. Following that, a forward stepwise multivariate logistic regression was used to evaluate the multiple risk factors associated with depression/anxiety after adjusting for significant sociodemographic variables. We chose logistic regression other than multiple linear regression because it was more likely to have practical significance. The odd ratio represented the extents that variables influenced the risk of depression or anxiety while the regression coefficient reflected the degrees that variables influenced the score of CES-D or GAD-7. Multicollinearity was assessed using the post-hoc variance inflation factor method. We planned to conduct a ridge regression if multicollinearity was found in the diagnostics.

## Results

Table 1 summarizes the associations between sociodemographic characteristics and depression/anxiety among MSM. The 547 MSM who completed the survey had a mean age of 30.5 years (range 17.3–65.3); 79.3% of the participants reported never having been married and 5.7% were divorced or widowed. Most (71.3%) reported being homosexual. HIV test outcomes were 5.9, 80.2, and 13.9% for positive, negative, and unknown, respectively. Binary logistic regression showed a significant relationship between the sociodemographic variable of education level and depression. Individuals with a college level or higher level of education had a lower level of depression (*ORu* = 0.579, 95% *CI* = 0.392–0.855). Binary logistic regression showed a significant relationship between the sociodemographic variable of income and anxiety. Individuals earning more than 6000 yuan per month had a lower level of anxiety (*ORu* = 0.494, 95% *CI* = 0.257–0.948).

**Table 1:** Sociodemographic characteristics and their associations with psychosocial variables for MSM participants in Shanghai (*N* = 547)

Socio-demographics	Number of the participants	Depression		Anxiety	
	*N* (col%)	*N* (*row*%)	*ORu* (95%*CI*)	*N* (*row*%)	*ORu* (95%*CI*)
*Age group (years)*					
< 25	148 (27.1)	41(27.7)	0.748(0.396–1.415)	20(13.5)	1.228(0.491–3.071)
25–40	337 (61.6)	107(31.8)	0.908(0.512–1.612)	40(11.9)	1.058(0.451–2.483)
> 40	62 (11.3)	21(33.9)	1	7(11.3)	1
*Employment status*					
Employed	447 (81.7)	137(30.6)	1	54(12.1)	1
Unemployed	100 (18.3)	32(32.0)	1.065(0.668–1.697)	13(13.0)	1.087(0.569–2.080)
*Highest education level*					
Senior high school or less	157 (28.7)	62(39.5)	1	21(13.4)	1
College degree or above	390 (71.3)	107(27.4)	0.579(0.392–0.855)^**^	46(11.8)	0.866(0.498–1.506)
*Current marital status*					
Married	82 (15.0)	24(29.3)	1	12(14.6)	1
Single	434 (79.3)	132(30.4)	1.056(0.629–1.773)	50(11.5)	0.760(0.385–1.499)
Divorced or widowed	31 (5.7)	13(41.9)	1.745(0.740–4.114)	5(16.1)	1.122(0.360–3.494)
*Income*					
< 3000	133 (24.3)	48(36.1)	1	23(17.3)	1
3000–6000	211 (38.6)	66(31.3)	0.806(0.510–1.274)	23(11.8)	0.643(0.348–1.187)
> 6000	203 (37.1)	55(27.1)	0.658(0.411–1.053)	19(9.4)	0.494(0.257–0.948)^*^
*Residence status*					
Local	147 (26.9)	38(25.9)	1	14(9.5)	1
Non-local	400 (73.1)	131(32.8)	1.397(0.914–2.135)	53(13.3)	1.451(0.779–2.703)
*Self-reported sexual orientation*					
Non-homosexual	157 (28.7)	44(28.0)	1	17(10.8)	1
Gay/ homosexual	390 (71.3)	125(32.1)	1.211(0.805–1.822)	50(12.8)	1.211(0.675–2.173)
*Have had a VCT*					
No	250 (45.8)	79(31.6)	1	30(12.0)	1
Yes	296 (54.2)	90(30.4)	0.946(0.657–1.361)	37(12.5)	1.048(0.627–1.752)
*HIV test outcome*					
Positive	24 (5.9)	5(20.8)	1	3(12.5)	1
Negative	330 (80.2)	96(29.1)	1.559(0.566–4.295)	35(10.6)	0.831(0.236–2.926)
Unknown	54 (13.9)	68(35.2)	2.067(0.739–5.781)	29(15.0)	1.238(0.347–4.419)

*ORu* Univariate odds ratio; 95% *CI* 95% confidence interval; *VCT* Voluntary HIV counseling and testing. ^*^p < 0.05, ^**^p < 0.01

### Psychosocial health conditions among men who have sex with men

Table 2 summarizes scores on the psychosocial health measures; 12.2% of participants showed high-level generalized anxiety disorder and 30.9% had experienced clinically significant depression symptoms.

**Table 2:** Frequency distributions of psychosocial variables for MSM participants (N = 547)

Psychosocial variables	Number	Percent%
*GAD-7-anxiety*		
Low level (score < 10)	480	87.8
High level (score > =10)	67	12.2
*CES-D-depression*		
Low level (score < 22)	378	69.1
High level (score > =22)	169	30.9

*GAD-7* Generalized Anxiety Disorder 7-item scale, *CES-D* Center for Epidemiologic Studies Depression scale

Table 3 shows that participants in the high-level group of anxiety or depression demonstrated more involuntary subordination and more perceived defeat, entrapment, submissive behavior, and unfavorable social comparisons.

**Table 3:** Involuntary subordination in different psychosocial groups

Variable	GAD-7-anxiety					CESD-depression				
	Low level		High level		*p*	Low level		High level		*p*
	(*N*_1_ = 480)		(*n*_1_ = 67)			(*N*_2_ = 378)		(*n*_2_ = 169)		
	*M*	*SD*	*M*	*SD*		*M*	*SD*	*M*	*SD*	
Defeat	16.74	5.588	23.955	5.695	< .001	15.286	4.808	22.852	5.32	< .001
Social Comparison	20.59	5.526	23.119	4.673	< .001	19.603	5.383	23.799	4.54	< .001
Submissive Behavior	21.552	4.366	25.105	3.993	< .001	21.143	4.257	23.876	4.377	< .001
Entrapment	19.063	5.949	26.746	5.218	< .001	17.759	5.406	25.024	5.477	< .001
Involuntary Subordination	77.944	16.965	98.925	14.715	< .001	73.791	15.296	95.55	14.335	< .001

*GAD-7* Generalized Anxiety Disorder 7-item scale, *CES-D* Center for Epidemiologic Studies Depression scale, *M* mean, *SD* Standard deviation

Table 4 shows the correlations between involuntary subordination and psychosocial variables. Scores on the ISQ and all four ISQ subscales were significantly correlated with depression and anxiety scores.

**Table 4:** Correlations between involuntary subordination and psychosocial variables

Variable	*M*	*SD*	1	2	3	4	5	6	7
1. Defeat	17.623	6.076	–						
2. Social Comparison	20.899	5.488	.415^**^	–					
3. Submissive Behavior	21.987	4.473	.616^**^	.122^**^	–				
4. Entrapment	20.004	6.380	.860^**^	.384^**^	.661^**^	–			
5. Involuntary subordination	80.514	18.057	.919^**^	.609^**^	.725^**^	.923^**^	–		
6. Anxiety	4.673	4.460	.507^**^	.261^**^	.331^**^	.536^**^	.521^**^	–	
7. Depression	17.159	10.712	.666^**^	.440^**^	.367^**^	.620^**^	.668^**^	.650^**^	–

*M* mean, *SD* Standard deviation. ^**^Significant Pearson correlation (*p* < 0.01, two-tailed)

### Association between involuntary subordination and depression

Table 5 summarizes the results of the binary regressions. After adjusting for education level, all four scales (DS, ES, SCRS, and SBS) and the ISQ showed a significant association with depression. Participants who reported a sense of defeat (*AOR* = 1.352, 95% *CI* = 1.280–1.428), entrapment (*AOR =* 1.272, 95% *CI* = 1.216–1.333), social comparison (*AOR* = 1.170, 95% *CI* = 1.123–1.220), submissiveness (*AOR* = 1.164, 95% *CI* = 1.108–1.223), and involuntary subordination were at increased risk of depression. The multivariate logistic regression showed that all the variables remained significantly associated with depression: defeat (*ORm* = 1.265, 95% *CI* = 1.166–1.372), social comparison (*ORm* = 1.119, 95% *CI* = 1.061–1.181), and entrapment (*ORm* = 1.132, 95% *CI* = 1.047–1.224). However, submissive behavior showed a reverse association with depression in the multivariate logistic regression (*ORm* = 0.897, 95% *CI* = 0.825–0.975). The multicollinearity diagnostics showed a variance inflation factor no greater than 10, so we concluded that the four involuntary subordination variables made independent contributions to the involuntary subordination score.

**Table 5:** The association between involuntary subordination and depression for MSM participants (*N* = 547)

Variable	*ORu* (95%*CI*)	*AOR* (95%*CI*)	*ORm* (95%*CI*)
Defeat	1.347(1.277–1.422)^**^	1.352(1.280–1.428)^**^	1.265(1.166–1.372)^**^
Social Comparison	1.175(1.128–1.224)^**^	1.170(1.123–1.220)^**^	1.119(1.061–1.181)^**^
Submissive Behavior	1.170(1.114–1.229)^**^	1.164(1.108–1.223)^**^	0.897(0.825–0.975)^*^
Entrapment	1.275(1.218–1.334)^**^	1.272(1.216–1.333)^**^	1.132(1.047–1.224)^**^
Involuntary subordination	1.113(1.092–1.136)^**^	1.115(1.093–1.138)^**^	

*CI* Confidence interval; *ORu* Univariate odds ratio, *AOR* Adjusted *OR*, odds ratios adjusted for highest education level; *ORm* Odds ratio obtained from forward stepwise multivariate logistic regression using significant variables from the univariate analysis as input. ^*^p < 0.05, ^**^p < 0.01

### Association between involuntary subordination and anxiety

Table 6 summarizes the results of the binary regressions. After adjusting for income level, all four scales (DS, ES, SCRS, and SBS) and the ISQ showed a significant association with anxiety. Participants who reported a sense of defeat (*AOR* = 1.236, 95% *CI* = 1.171–1.304), social comparison (*AOR* = 1.089, 95% *CI* = 1.039–1.145), submissiveness (*AOR* = 1.225, 95% *CI* = 1.142–1.314), entrapment (*AOR* = 1.256, 95% *CI* = 1.188–1.329), and involuntary subordination (*AOR* = 1.086, 95% *CI* = 1.064–1.110) were at increased risk of depression. The multivariate logistic regression showed that only two variables remained significantly associated with anxiety: defeat (*ORm* = 1.091, 95% *CI* = 1.004–1.185) and entrapment (*ORm* = 1.174, 95% *CI* = 1.079–1.278).

**Table 6:** The association between involuntary subordination and anxiety for MSM participants (*N* = 547)

Variable	*ORu* (95%*CI*)	*AOR* (95%*CI*)	*ORm* (95%*CI*)
Defeat	1.232(1.170–1.298)^**^	1.236(1.171–1.304)^**^	1.091(1.004–1.185)^*^
Social Comparison	1.091(1.039–1.146)^**^	1.089(1.039–1.145)^**^	
Submissive Behavior	1.226(1.143–1.314)^**^	1.225(1.142–1.314)^**^	
Entrapment	1.253(1.186–1.324)^**^	1.256(1.188–1.329)^**^	1.174(1.079–1.278)^**^
Involuntary subordination	1.084(1.063–1.107)^**^	1.086(1.064–1.110)^**^	

*ORu* Univariate odds ratio, *AOR* Adjusted *OR*, odds ratios adjusted for income; *ORm* Odds ratio obtained from forward stepwise multivariate logistic regression using significant variables from the univariate analysis as input. ^*^p < 0.05, ^**^p < 0.01

## Discussion

Our findings indicated a high prevalence of psychosocial problems among MSM in Shanghai, China. Almost one third of the participants (30.9%) suffered from depression; the prevalence of depression in the general adult population in China is 2.06%. The findings also showed that anxiety was more prevalent in our sample (12.2%) than in the general population in China (1.32%) [31]. The results of the current study indicate that the MSM population experiences poor mental health. As expected, all ISQ components were related to depression and anxiety among MSM.

The significant associations found here between sociodemographic variables and depression and anxiety support previous findings. MSM who have a higher education level are less likely to have depression symptoms. Education improves well-being in men, because it increases work creativity and a sense of control and provides payoffs, such as feelings of dominance and earning capacity [34]. We found that income was associated with anxiety disorders; a pattern consistent with previous study findings [10]. Previous research indicates that low income is linked to distress, because it functions as an indirect proxy for social rank [9, 52].

Involuntary subordination manifests as clinical depression and anxiety disorders when individuals cannot accept their new social position following failure in competition [38, 39]. Involuntary subordination, as a reflection of social strata at the community level, affects psychological and emotional factors at an individual level. Recent research has clarified the role of social adaptive functions in psychological problems. Depression has been viewed as a mechanism that reduces social risk [3] and social loss [22], and solves social problems through rumination, agitation, and the seeking of social support [51].

All the involuntary subordination variables were associated with both depression and anxiety after adjusting for sociodemographic factors. This could be explained by the relatively high comorbidity of depression and anxiety and the overlap between the symptomatology and neurology of these disorders [30]. Allan and Gilbert have demonstrated that poor social comparison is associated with higher levels of interpersonal sensitivity and depression in both nonclinical student and psychiatric inpatient groups [16]. Sturman and Mongrain [43]. have shown that internal and external entrapment account for about 50% of the variance in self-reported levels of depression. Depressed individuals have a strong desire to escape from aversive thoughts or external circumstances and perceive their situation as uncontrollable. One study found that submissive behavior (labeled as “subassertive”) was associated with various psychological problems (e.g., depression and social anxiety) [21]. One systematic review [46] has confirmed that defeat and entrapment both play a central role in depressive symptoms, anxiety, and suicidality.

The present findings showed that defeat, entrapment, low perceived status, and submissiveness were still significantly associated with depression after adjusting for sociodemographic factors. However, only defeat and entrapment were significantly associated with anxiety in both the univariate and multivariate regression models. Although anxiety and depression may share common evolutionary origins [29], some research indicates that anxiety and depression have different functions in the involuntary subordination process. Whereas depression helps to avoid future defeat, anxiety promotes reconciliation, increases sensitivity to possible threats, and focuses on future attacks that would result in loss of status [37]. The perception of defeat begins with an initial evaluation of the current situation followed by a perception of entrapment (and a decision whether to escape or not). Submissiveness and low perceived status suggest that positive self-appraisals such as self-worth and adequacy are absent, whereas defeat and entrapment indicate that negative cognitions are overactivated. Research suggests that depression is a complex combination of high negative affectivity and low positive affectivity, but that anxiety is only correlated with high negative affectivity [50]. Moreover, we found that submissive behavior was a protective factor for depression in the multivariate regression models. It may be that submissive behavior plays a vital role in group cohesion and the control of agonistic behavior [25], maintains the individual’s social position, and redirects behavior toward more productive pursuits.

These findings could form the basis of a new, integrated, and holistic approach to the identification of high-risk groups and the development of interventions for anxiety and depression among MSM. MSM may improve their social status and increase their self-efficacy and resilience through targeted interventions. Further research like a prospective cohort study can be designed to validate the exact causal relationship between involuntary subordination, anxiety and depression. Randomized controlled trials can be designed to explore intervention strategies for these common mental disorders among MSM. In addition, an open and tolerant social environment is required for MSM and other sexual minority groups.

### Limitations

Several study limitations should be acknowledged in drawing conclusions from the results. First, because this was a cross-sectional study, causality cannot be inferred from these results. That is, we cannot determine whether involuntary subordination causes depression and anxiety. It remains for future longitudinal research to determine whether and how involuntary subordination is involved in the onset, maintenance, and recovery from depression and anxiety. Second, the snowball sampling technique we used may have caused selection bias. However, this method is commonly used in targeting hard-to-reach populations. More studies are needed with larger sample sizes to validate our conclusions. Finally, all data were self-reported and participants’ responses may not have been completely honest; biases in self-reported data are inevitable.

## Conclusions

Our findings provide evidence of the prevalence of specific psychosocial problems among MSM in Shanghai, China. MSM are more likely to be depressed or anxious. This may have implications for mental healthcare services and social support for MSM. This study used an evolutionary framework to understand the relationship between mental disorders and involuntary subordination. As such, it offers a new, innovative perspective on the association between individual psychological problems and social rank problems.

## Data Availability

The datasets used and analyzed during the current study are available from the corresponding author on reasonable request.

## Abbreviations

CDC: Center for Disease Control and Prevention
CES-D: Center for Epidemiologic Studies Depression scale
CI: Confidence interval
DS: Defeat Scale
ES: Entrapment Scale
GAD-7: Generalized Anxiety Disorder 7-item scale
HIV: Human immunodeficiency virus
ISQ: Involuntary Subordination Questionnaire
M: Mean
MSM: Men who have sex with men
N: Number
NGO: Non-governmental organization
OR: Odds ratio
SBS: Submissive Behavior Scale
SCRS: Social Comparison Rating Scale
SD: Standard deviation
VCT: Voluntary human immunodeficiency virus counseling and testing
